# Evolutionary patterns and research frontiers in neoadjuvant immunotherapy: a bibliometric analysis

**DOI:** 10.1097/JS9.0000000000000492

**Published:** 2023-05-20

**Authors:** Shitao Jiang, Yaoge Liu, Han Zheng, Lei Zhang, Haitao Zhao, Xinting Sang, Yiyao Xu, Xin Lu

**Affiliations:** Department of Liver Surgery, State Key Laboratory of Complex Severe and Rare Diseases, Peking Union Medical College Hospital, Chinese Academy of Medical Sciences and Peking Union Medical College, Beijing, China

**Keywords:** bibliometrics, CiteSpace, frontiers, neoadjuvant immunotherapy, VOSviewer

## Abstract

Research has shown that neoadjuvant immunotherapy may provide more significant clinical benefits to cancer patients undergoing surgery than adjuvant therapy. This study examines the development of neoadjuvant immunotherapy research using bibliometric analysis. As of 12 February 2023, articles on neoadjuvant immunotherapy in the Web of Science Core Collection were collected. Co-authorship and keyword co-occurrence analyses and visualizations were performed using VOSviewer, while CiteSpace was used to identify bursting keywords and references. The study analyzed a total of 1222 neoadjuvant immunotherapy publications. The top contributors to this field were the United States, China, and Italy, and the journal with the most publications was Frontiers in Oncology. Francesco Montorsi had the highest H-index. The most common keywords were ‘immunotherapy’ and ‘neoadjuvant therapy’. The study conducted a bibliometric analysis of over 20 years of neoadjuvant immunotherapy research, identifying the countries, institutions, authors, journals, and publications involved in this field. The findings provide a comprehensive overview of neoadjuvant immunotherapy research.

## Introduction

Highlights

- This study presents the first bibliometric analysis in the field of neoadjuvant immunotherapy.
- Burst detection revealed that applying neoadjuvant immunotherapy for urothelial carcinoma and radical cystectomy had become a hot topic.
- The United States is the leading country in articles, researchers, and institutions researching neoadjuvant immunotherapy, with China following closely behind.

Neoadjuvant immunotherapy has emerged as a promising approach for treating various types of cancer, including melanoma^1^ and breast cancer^2^. It involves administering immunotherapeutic agents before surgical resection to enhance the host immune response and reduce the primary tumor size^3^. The use of neoadjuvant immunotherapy in cancer treatment has gained significant attention in recent years, and there is a growing body of literature on the subject. An in-depth bibliometric study of publications, countries, institutions, journals, authors, and keywords is still required.

Bibliometrics is a field of study that uses quantitative and statistical methods to analyze the production and dissemination of research literature. It involves collecting, organizing, and analyzing bibliographic data, such as the number of citations, co-authorship patterns, and publication venues^4^. Bibliometrics has several advantages, including the ability to identify and quantify the impact of research, provide evidence-based assessments of scientific productivity, and track the dissemination and influence of study over time. Bibliometrics can also support the identification of research trends, emerging fields, and collaborations and inform strategic planning and resource allocation in research organizations^5^. With the growing volume of scientific literature and the increasing importance of research impact, bibliometrics will continue to play a significant role in evaluating and assessing research.

This bibliometric study aims to provide a comprehensive overview of the current knowledge and understanding of neoadjuvant immunotherapy. This study addresses the following research question: What is the current knowledge and experience of neoadjuvant immunotherapy in cancer treatment? A thorough bibliometric analysis was conducted to assess the trends, findings, and gaps in the existing research on neoadjuvant immunotherapy. This research revealed that neoadjuvant immunotherapy had shown promising results in preclinical and clinical trials, with some studies demonstrating improved outcomes compared to standard adjuvant therapies. However, the literature also highlights the need for further research to fully understand the mechanisms underlying neoadjuvant immunotherapy’s efficacy and optimize treatment protocols. This study will fill the gap in the existing literature by synthesizing the present findings and trends in the field, providing a comprehensive overview of the current state of knowledge and understanding about neoadjuvant immunotherapy for researchers, clinicians, and policymakers.

## Method

### Data source and literature search strategy

Web of Science was selected as the primary database for this study due to its comprehensive coverage of over 12 000 academic journals and its frequent usage by researchers. When compared to other databases such as Scopus, Medline, and PubMed, Web of Science provides the most comprehensive and reliable bibliometric analysis^6^. The relevant articles in the Web of Science Core Collection (WoSCC) were searched and exported on 12 February 2023, using all database versions. All authors agreed on the search strategy following consultations with senior literature search experts, with the following criteria: (TS = (Neoadjuvant) AND (TS = (Immunotherapy) OR (TS = (Immunotherapies)). To facilitate further analysis of literature content, only regular articles written in English were included. A complete record and cited references were then extracted from relevant publications, saved in plain text format, for further research. To further compare the development patterns and frontier topics of neoadjuvant immunotherapy in different cancer types, we conducted an additional investigation on 25 April 2023, examining the publication status of neoadjuvant immunotherapy articles for the top 5 cancer types by frequency. The keyword frequency, search strategy, and the number of articles are displayed in Table S2, Supplemental Digital Content 1, http://links.lww.com/JS9/A537. The remaining limitations are consistent with the primary search strategy mentioned above.

### Software for bibliometric analysis

This study used R version 4.0.1^7^, VOSviewer^8^, and CiteSpace^9^ as the software tools for performing bibliometric analysis. We calculated the frequency of collaboration between countries using the Bibliometrix R package version 4.0.0^10^. The number of publications, citations, and keyword frequency were calculated using VOSviewer. With the software’s embedded clustering algorithm, co-occurrence networks of essential keywords from scientific literature were constructed and visualized^11^. The co-authorship and co-occurrence analysis was the primary focus of this study. This tool was used to analyze country, institution, and author collaborations.

To identify highly cited references and keywords that experienced significant citation increases during a specific period, CiteSpace was used.

Through the online bibliometrics website (https://bibliometric.com/), we were able to visualize international collaborations between countries. The number of publications was analyzed using an exponential growth function in Excel^12^.

## Result

### Overview of Publication Status

**Figure 1:**
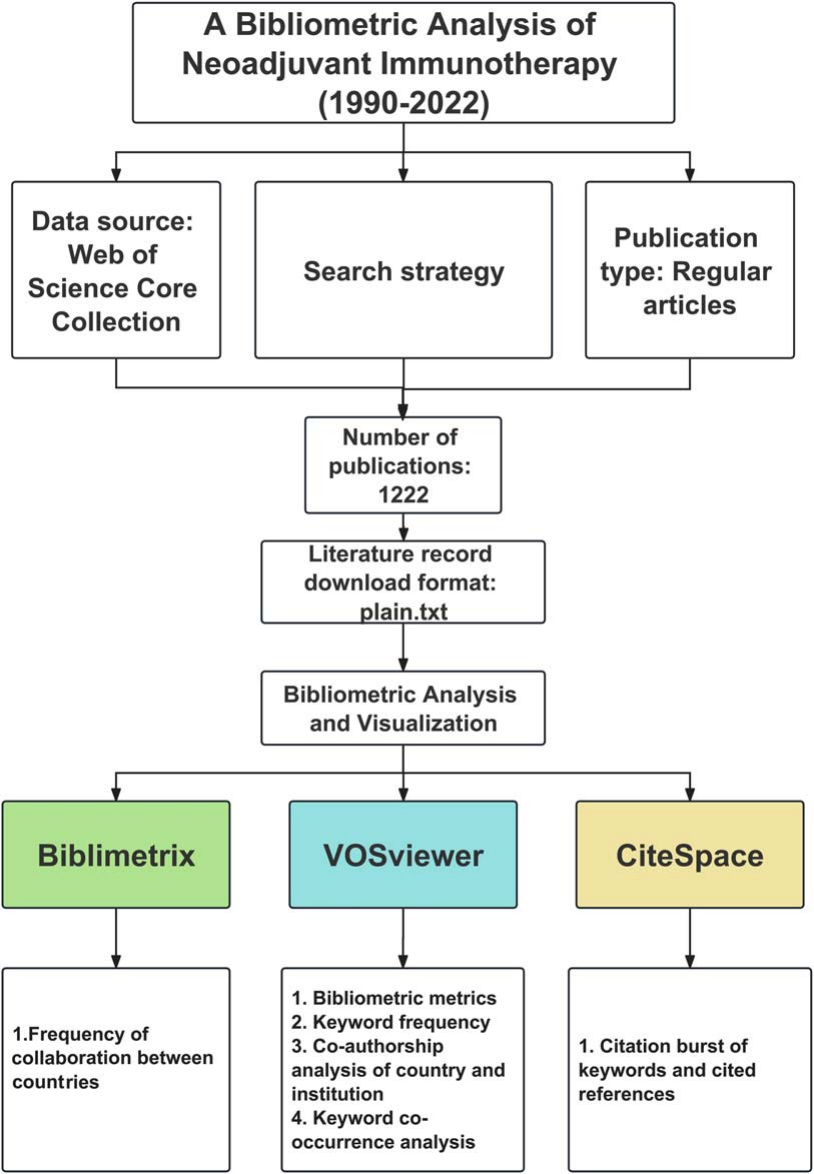
Flow-chart of the study.

**Figure 2:**
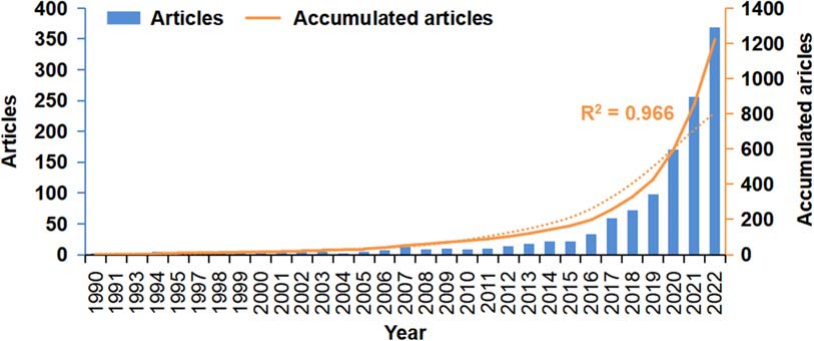
Number of publications per year and the cumulative number.

As shown in Figure 1, 1222 conventional articles on neoadjuvant immunotherapy were included in this study. Figure 2 displays the annual and cumulative publication counts related to neoadjuvant immunotherapy. From 1 publication in 1990 to 10 publications in 2011, the cumulative number has steadily increased. Immune checkpoint inhibitors (ICIs) subsequently led to a rapid increase in publications over the following 11 years, reaching 1222 in 2022. An exponential growth function was then used to evaluate the relationship between cumulative publications and publication year, which matched the trend in the cumulative number of publications (R^2^=0.966). This strong correlation suggests that neoadjuvant immunotherapy has experienced significant growth and development, particularly in the era of ICIs.

### Analysis of national publication counts

**Figure 3:**
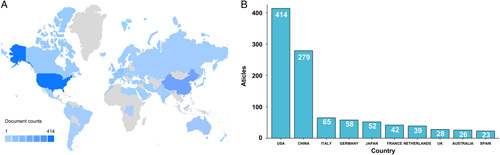
Each country’s contribution to the neoadjuvant immunotherapy.

National publication counts were analyzed to investigate the countries/regions contributing the most in this field. In Figure 3, the United States ranks first with 414 publications, followed by China (279), Italy (65), Germany (58), and Japan (52). The remaining countries/regions have fewer than 50 publications.

As part of our investigation, we visualized the collaborations among countries/regions in Figure S1, Supplemental Digital Content 2, http://links.lww.com/JS9/A538. According to the results, the US leads the way in neoadjuvant immunotherapy research. The most frequent collaborations are between the US and China and between the US and Italy (with a frequency of 47). The following are the most frequent collaborators are: Germany (frequency=43), France (frequency=35), and the UK (frequency=34). The collaborators from these countries are all from the US. Thirty-four countries have published five or more articles (34/56). We conducted a co-authorship analysis of all publications from the 34 countries above (Figure S2, Supplemental Digital Content 2, http://links.lww.com/JS9/A538) to investigate the collaboration between countries. The size of the circles in the clustering network and the time-overlapping network represent the number of publications. The color of the circles indicates the collaboration strength of the research groups in the clustering network. The colors of the circles represent the average publication year for each country in a specific area of research in the time-overlapping network. As shown in Figure S2A, Supplemental Digital Content 2, http://links.lww.com/JS9/A538, the 34 countries formed five clusters. The blue cluster, which includes most countries, has seven countries. In Figure S2B, Supplemental Digital Content 2, http://links.lww.com/JS9/A538, the US was an early pioneer in neoadjuvant immunotherapy, while Chinese researchers’ studies in this field are relatively recent.

### Analysis of institution publications

**Figure 4:**
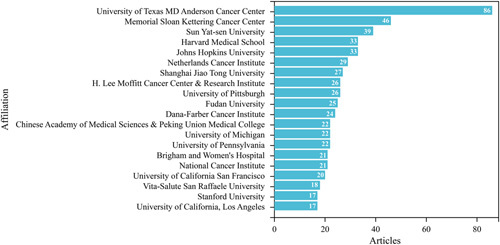
The top 20 institutions with the most publications in the field of neoadjuvant immunotherapy.

To explore institutions’ contributions to neoadjuvant immunotherapy, the number of publications from various institutions was analyzed. Neoadjuvant immunotherapy research was conducted at approximately 2296 institutions worldwide. As shown in Figure 4, 14 research institutions are from the US, four from China, one from Italy, and one from the Netherlands. With 86 articles published, the University of Texas MD Anderson Cancer Center ranked first.

To further investigate collaboration between institutions, we performed a co-authorship analysis of all publications. Supplementary Figure 3A, Supplemental Digital Content 2, http://links.lww.com/JS9/A538 shows that 68 institutions published at least 10 papers. These 68 institutions formed five clusters, with the red cluster being the largest, consisting of 21 institutions, mainly from China. Several American research institutions, led by Johns Hopkins University, contributed significantly to neoadjuvant immunotherapy’s early development. In contrast, multiple Chinese research institutions became more involved in neoadjuvant immunotherapy research after 2020.

### Analysis of publication quantity and journal impact

The study included 1222 articles published in 405 journals. Table 1 lists the top 10 journals ranked by publication quantity and their latest 2022 impact factors (IF)^13^. Seven top 10 journals were in the first quartile (Q1) of the Journal Citation Reports (JCR). There are three publishers from the US, UK, and Switzerland, but only one publisher from China.

**Table 1:** Top 10 journals in the field of neoadjuvant immunotherapy.

Rank	Source	Article	Country	IF	H-index	JCR-c
1	Frontiers in Oncology	54	Switzerland	5.738	53	Q2
2	The Journal for ImmunoTherapy of Cancer	48	UK	12.469	46	Q1
3	Clinical Cancer Research	43	USA	13.801	61	Q1
4	Cancers	39	Switzerland	6.575	54	Q1
5	Frontiers in Immunology	37	Switzerland	8.786	84	Q1
6	Oncoimmunology	29	USA	7.723	26	Q1
7	BMC Cancer	28	UK	4.638	31	Q2
8	Cancer Immunology Immunotherapy	28	USA	6.63	30	Q1
9	Journal of Translational Medicine	17	UK	8.44	46	Q1
10	Annals of Translational Medicine	15	China	3.616	32	Q3

### Author impact analysis

A total of 10 825 authors participated in research on neoadjuvant immunotherapy. Table 2 shows Andrea Necchi to be the most productive author, with 18 articles published and a 39 H-index. Following closely behind were Francesco Montorsi (14 articles, H-index=110) and Alberto Briganti (13 articles, H-index=81).

**Table 2:** Top 10 authors in the field of neoadjuvant immunotherapy.

Rank	Author	Article	H-index
1	Andrea Necchi	18	39
2	Francesco Montorsi	14	110
3	Alberto Briganti	13	81
4	Paolo A. Ascierto	12	83
5	Christian Blank	12	63
6	Jennifer A. Wargo	12	83
7	Marco Bandini	11	41
8	Gallina Andrea	11	51
9	Lajos Pusztai	11	74
10	Daniele Raggi	11	19

Researchers’ collaborative relationships are illustrated in Supplementary Figure 4A, Supplemental Digital Content 2, http://links.lww.com/JS9/A538. Circle size represents the number of publications, and color represents cluster. One hundred twenty-two authors with five or more articles were grouped into 14 clusters. Six clusters were located outside the more prominent community consisting of eight clusters. There was no collaboration between these six scattered clusters and the larger clusters, indicating that collaboration between research teams/laboratories conducting research related to neoadjuvant immunotherapy needs to be strengthened. The time-overlapping network of clustering results is shown in Supplementary Figure 4B, Supplemental Digital Content 2, http://links.lww.com/JS9/A538. We observed that researchers from China are forming a new research network on neoadjuvant immunotherapy. National and institutional collaborations are one of the future directions due to the inadequate collaboration between different research groups.

### Research hotspot analysis

#### Most cited publications

It is possible to evaluate the most cited articles based on the frequency of citations in that field. In Supplementary Table 1, Supplemental Digital Content 9, http://links.lww.com/JS9/A545, we list the top 10 most cited publications, with all articles cited over 300 times. There was one article published in 2018 that has received the most citations: ‘Neoadjuvant PD-1 Blockade in Resectable Lung Cancer’^14^. The study reported that neoadjuvant therapy with nivolumab had few side effects, did not delay surgery, and induced complete pathological responses in 45% of resected tumors. The New England Journal of Medicine also published the second most cited publication. Schmid *et al*. found that in early triple-negative breast cancer (TNBC) patients, patients who received pembrolizumab plus neoadjuvant chemotherapy had a significantly higher rate of complete pathological response than who received placebo plus neoadjuvant chemotherapy^15^. These two studies provide strong evidence for the clinical application of neoadjuvant immunotherapy.

#### Analysis of citation bursts

The top 25 most cited references are illustrated in Supplementary Figure 5, Supplemental Digital Content 2, http://links.lww.com/JS9/A538. A burst is when a publication receives a significantly higher number of citations than usual, lasting at least two years^9^. The blue line represents the observation period from 1990 to 2022, while the red line indicates the burst time. The article ‘Safety, Activity, and Immune Correlates of Anti–PD-1 Antibody in Cancer’, published in the New England Journal of Medicine, has the highest citation burst value (citation burst=14.08) between 1990 and 2022^16^. Additionally, 11 articles are still experiencing citation bursts, including ‘Durvalumab after Chemoradiotherapy in Stage III NonSmall-Cell Lung Cancer’, which has the highest burst value of 11.24. This article was published in the New England Journal of Medicine in 2017 and reports on the safety and efficacy of using Durvalumab after chemotherapy in stage III nonsmall-cell lung cancer^17^. The results show that the progression-free survival is significantly longer with Durvalumab than with a placebo, and the safety between the two groups is similar. Necchi *et al*. reported that neoadjuvant pembrolizumab therapy resulted in a pT0 status in 42% of patients and was safe in patients with muscle-invasive urothelial bladder carcinoma (MIBC). This study suggests that pembrolizumab may be a reliable neoadjuvant therapy for MIBC when restricted to patients with PD-L1-positive or high tumor mutation burden tumors^18^. In the future, such research topics may continue to be popular and become a potential frontier area of neoadjuvant immunotherapy research.

#### Frequency and clustering analysis of keywords

**Figure 5:**
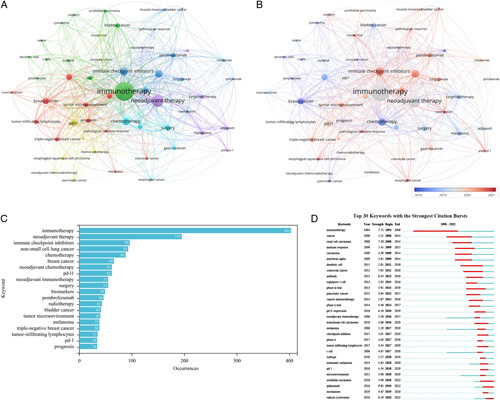
Research hotspots on neoadjuvant immunotherapy (A). keyword co-occurrence network; (B). time-overlapping co-occurrence analysis network of keywords; (C). a list of the 20 most frequently used keywords; (D). the 20 keywords with the strongest citation bursts).

Among 2027 keywords, 54 met the threshold of 10 occurrences, and were analyzed. If these keywords had similar meanings, they were merged. Figure 5A shows the network visualization of these keywords. Keyword frequency is reflected in the size of the nodes, while relationship strength is reflected in the distance between them^8^. In order to reflect the critical themes in neoadjuvant immunotherapy research, the 54 keywords were grouped into six clusters. Closely related keywords were clustered together. Group 1, represented in red, focused on the application of neoadjuvant chemotherapy in different cancers, such as ‘breast cancer’, ‘colorectal cancer’, and ‘ovarian cancer’. Group 2, represented in green, focused on various treatment methods for bladder and urothelial carcinoma, with keywords such as ‘cisplatin’, ‘cystectomy’, ‘immunotherapy’, and ‘PD-1’. Some terms, such as ‘survival’, were also included in Group 2. Group 3, represented in blue, focused on the clinical application of neoadjuvant immunotherapy, with keywords such as ‘neoadjuvant immunotherapy’, ‘lung cancer’, ‘muscle-invasive bladder cancer’, and common ICIs. The yellow Group 4 focused on the clinical application of neoadjuvant chemoradiotherapy combined with immunotherapy, mainly involving ‘pancreatic cancer’, ‘prostate cancer’, ‘esophageal squamous cell carcinoma’, and ‘rectal cancer’. The purple Group 5 mainly included adjuvant and neoadjuvant therapy for renal cell carcinoma. The sixth light blue cluster mainly included ‘esophageal cancer’, ‘gastric cancer’, ‘chemotherapy’, ‘surgery’, and ‘radiotherapy’, which seemed to be related to traditional treatment methods. Figure 5B shows the visualization of the time-overlapping of keywords. Early-appearing keywords are displayed in blue, while recent keywords are displayed in red. Early research mainly focused on ‘chemotherapy’, ‘renal cell carcinoma’, and ‘breast cancer’. Recent studies have concentrated on topics such as ‘lung cancer’, ‘esophageal squamous cell carcinoma’, ‘complete pathologic response’, ‘tumor microenvironment’, and ‘immune checkpoint inhibitors’. Figure 5C displays the top 20 keywords sorted by frequency, with ‘immunotherapy’being the most frequently used keyword, appearing 403 times, followed by ‘neoadjuvant therapy’ (*N*=195) and ‘immune checkpoint inhibitors’ (*N*=96). ‘Neoadjuvant immunotherapy’ appeared 55 times, ranking ninth. Among the top 20 keywords, cancer types that appeared included ‘non-small cell lung cancer (NSCLC)’ (*N*=93), ‘breast cancer’ (*N*=66), ‘bladder cancer’ (*N*=41), ‘melanoma’ (*N*=38) and ‘triple-negative breast cancer’ (*N*=38).

#### Comparison of neoadjuvant immunotherapy research across cancer types

To compare the progress of neoadjuvant immunotherapy development across different cancer types, we conducted a bibliometric analysis of the top five cancer types by keyword frequency. In summary, bibliometric analysis indicates that the research popularity of neoadjuvant immunotherapy varies among cancer types, with melanoma and NSCLC demonstrating the most significant growth in publications and citations. In comparison, bladder cancer, breast cancer, and TNBC have experienced relatively slower progress. This information is valuable for oncologists and researchers to identify areas of opportunity and focus their efforts on advancing neoadjuvant immunotherapy research across all cancer types. Table S3, Supplemental Digital Content 3, http://links.lww.com/JS9/A539 presents the top 10 countries, institutions, journals, and authors regarding publication volume for each cancer type. The United States is leading in neoadjuvant immunotherapy for breast, bladder, melanoma, and TNBC. China ranks first with 91 published articles on neoadjuvant immunotherapy for NSCLC. The University of Texas System has the highest number of publications in neoadjuvant immunotherapy research for NSCLC, breast cancer, and melanoma, consistent with the overall research findings. The journals with the most publications in NSCLC, breast cancer, bladder cancer, melanoma, and TNBC are Frontiers in Oncology, Clinical Cancer Research, Urologic Oncology: Seminars and Original Investigations, Journal of Translational Medicine, and Clinical Cancer Research, respectively. In NSCLC, the most active author is Boris Sepesi, with six published articles. Lajos Pusztai has published 11 articles on neoadjuvant immunotherapy for breast cancer and six on neoadjuvant immunotherapy for TNBC, making him the most active author in both fields. Andrea Necchi is the most active author in bladder cancer neoadjuvant immunotherapy research, with 15 articles published. In melanoma research, Paolo A. Ascierto leads the field with 11 articles.

To further understand the application of neoadjuvant immunotherapy across different cancer types, we analyzed the citation frequency of the included articles. Tables S4 through S8, Supplemental Digital Content 4, http://links.lww.com/JS9/A540, Supplemental Digital Content 5, http://links.lww.com/JS9/A541, Supplemental Digital Content 6, http://links.lww.com/JS9/A542, Supplemental Digital Content 7, http://links.lww.com/JS9/A543, Supplemental Digital Content 8, http://links.lww.com/JS9/A544 present the top 10 most cited articles in neoadjuvant therapy research for NSCLC, breast cancer, bladder cancer, melanoma, and TNBC.

#### Analysis of keywords bursts

In Figure 5D, we present the top 30 keywords with the strongest citation bursts lasting at least one year. The keywords ‘immunotherapy’ (1994–2009) received the most sustained attention. However, keywords such as ‘radical cystectomy’ (2020–2022), ‘ipilimumab’ (2020–2022), and ‘urothelial carcinoma’ (2020–2022) have recently been used, indicating that future research will focus on these keywords.

## Discussion

Bibliometric methods were used to analyze the growth pattern of neoadjuvant immunotherapy-related research from 1990 to 2022. There are two stages of neoadjuvant immunotherapy-related research growth based on whether the number of publications exceeds 10 per year for two consecutive years. Before 2011, there was a slow growth stage, except for 12 publications in 2007. The number of publications in other years was at most 10. Since 2016, neoadjuvant immunotherapy-related research has entered a rapid growth stage, with more than 10 publications per year. By 2022, the annual publication volume has reached 1222, indicating that neoadjuvant immunotherapy-related research has entered a phase of rapid development. The potential reason may be that with the popularity of ICIs in cancer treatment, people began to realize that moving ICIs treatment forward may favor patients^3,19^. Therefore, research institutions have continued to increase their support for neoadjuvant immunotherapy-related research, and research funding has continued to increase, promoting the rapid development of this field.

A total of 1026 articles were published by the top 10 countries, accounting for 83.96% of all articles. The US and China dominate the number of publications among the 10 countries. Furthermore, US-centered international cooperation occupies eight positions in the 10 countries with the highest frequency of cooperation. The above findings confirm the US’s critical contributions and leading position in neoadjuvant immunotherapy research, which may result from the United States’ national economic conditions and high medical investment levels. This field will benefit from extensive international cooperation, which will improve the overall standard of research.

Fourteen of the top 20 institutions are in the US, similar to the distribution of publication numbers by country. Despite China’s second-place ranking in publication numbers, only four institutions make the top 20. Italy ranked third in the number of publications and has only one institution in the top 20, ranking 17th. In contrast, a Netherlands institution ranks sixth with 29 papers. These studies are mainly based on international cooperation, indicating that seeking extensive cooperation between institutions may be crucial to improving research competitiveness under economic or resource limitations.

A peer-reviewed journal is essential to academic publishing. Core journals often publish the necessary research in the field. Researchers can identify potential journals to submit their manuscripts to based on the number of journal publications in the neoadjuvant immunotherapy field. Frontiers in Oncology has the most publications, with 54. The journal with the highest impact factor is Clinical Cancer Research (IF 13.801), followed by The Journal for ImmunoTherapy of Cancer (IF 12.469). Impact factor and JCR are commonly used indicators to evaluate the influence of journals. JCR divides all journals into four quartiles (Q1–Q4) based on their IF. In the top 10 journals by paper count, Q1 journals account for 70%. Moreover, despite China’s significant contributions to neoadjuvant immunotherapy research, Asian publishers are underrepresented in the top 10 journals. There is a need to establish and develop internationally influential journals in Asia.

This study aims to answer a question regarding the research hotspots that were widely studied by researchers over a certain period of time. Citation count can serve as one of the indicators of the academic influence of a publication^20^. Highly cited publications often represent the fundamental themes in a research field. Research hotspots can be identified by calculating citation counts and identifying highly cited publications. In this study, the 10 most frequently cited publications were published between 2013 and 2020 and mainly focused on neoadjuvant immunotherapy’s mechanism and clinical application.

In 2013, Klug *et al*. suggested that low-dose irradiation of human pancreatic cancer with new adjuvant local gamma rays can normalize abnormal blood vessels and effectively recruit tumor-specific T cells in the tumor area. The authors further verified T-cell-mediated antitumor immune response and extended survival in otherwise immune refractory spontaneous and xenotransplant mouse tumor models^21^. Although this study does not directly confirm that neoadjuvant immunotherapy provides a survival benefit for pancreatic cancer patients, it provides an essential theoretical basis for neoadjuvant immunotherapy and boosts the investigators’ confidence.

In 2016, Loi *et al*. analyzed tumor-infiltrating lymphocytes (TILs)’ clinical and molecular characteristics in residual lesions of TNBC after neoadjuvant chemotherapy. Their research aimed to develop a better understanding of the complex interactions between TILs and TNBC in order to improve treatment strategies. They explored a treatment strategy combining MEK inhibitors and PD-1/PD-L1 targeted immunotherapy in a mouse model. The authors pointed out that activating the Ras-MAPK pathway could promote immune escape in TNBC. Their findings suggest that a combined approach targeting MEK and PD-L1 might effectively counteract this immune escape, potentially leading to improved outcomes for TNBC patients^22^. In addition, Liu *et al*. used a mouse model of spontaneous metastatic breast cancer to demonstrate that neoadjuvant therapy was significantly more effective than adjuvant therapy^23^. These studies highlight the potential of neoadjuvant immunotherapy as a promising treatment strategy for TNBC patients, particularly when combined with targeted therapies such as MEK inhibitors. By building on these foundational findings, future research can continue to refine and optimize treatment approaches for TNBC, ultimately improving patient outcomes and quality of life.

In 2018, Forde *et al*. published an article titled ‘Neoadjuvant PD-1 Blockade in Resectable Lung Cancer’ in the New England Journal of Medicine, which had the highest number of citations in this research. In this study, the authors administered PD-1 inhibitor nivolumab to adults with previously untreated, operable early (stage I, II, or IIIA) NSCLC. The results showed that the side effects of neoadjuvant therapy with nivolumab were acceptable and unrelated to the delay of surgery. Nine of the 20 resected tumors showed a complete pathological response (45%)^14^. As higher complete response rates are associated with a better prognosis, this study’s results further increase the researchers’ confidence to conduct clinical studies on neoadjuvant immunotherapy. Subsequently, researchers have achieved breakthroughs in patients with glioma^24^, TNBC^15^, and colon cancer^25^, confirming the safety and effectiveness of neoadjuvant immunotherapy from multiple perspectives and cancer types. However, a clinical study conducted by Blank *et al*. suggests that although neoadjuvant immunotherapy seems promising, the current regimen (ipilimumab 3 mg/kg and nivolumab 1 mg/kg, two courses before and two courses after surgery) induced high toxicity in resectable stage III melanoma patients, with 9/10 patients experiencing one or more grade 3/4 adverse events. Therefore, further research is needed to maintain efficacy while reducing toxicity^26^. Overall, while the results from numerous studies demonstrate the potential of neoadjuvant immunotherapy in treating different cancers, continued research is necessary to refine treatment regimens and ensure the best possible outcomes for patients.

In 2020, Helmink reported a critical study emphasizing the importance of focusing on immune cells and structures beyond T cells to develop new biomarkers and immune-enhancing strategies. Helmink *et al*. found that B-cell biomarkers were differentially expressed in tumors of immune responders and nonresponders, indicating that B cells and their products may play a critical role in the antitumor immune response^27^. By integrating B-cell biomarkers and further exploring the roles and interactions of various immune cells within the tumor microenvironment, researchers may enhance the overall efficacy of cancer immunotherapy and contribute to developing more effective, personalized treatment strategies.

Since keywords reflect the core content of a study, co-occurrence analysis can identify high-frequency keywords that appear in different studies, thus helping researchers to quickly grasp research hotspots. In this study, the most frequent keywords were ‘immunotherapy’ and ‘neoadjuvant therapy’. The frequency of ‘neoadjuvant immunotherapy’ was 55 times. Furthermore, ‘biomarkers’ were another frequently appearing keyword. Studies have shown that biomarkers can predict the efficacy of ICIs, disease progression, and recurrences^28–30^. However, research on biomarkers related to neoadjuvant immunotherapy is rare. Since some patients may experience disease progression after neoadjuvant immunotherapy or lose the opportunity for surgery due to immune-related adverse events, screening for patient groups with potential benefits for neoadjuvant immunotherapy is critical^31,32^. Exploring biomarkers related to the efficacy of neoadjuvant immunotherapy through various methods can be an effective tool for maximizing individual treatment outcomes. In conclusion, co-occurrence analysis of high-frequency keywords in research studies can highlight focus areas, such as neoadjuvant immunotherapy and biomarkers. Expanding our understanding of biomarkers related to the efficacy of neoadjuvant immunotherapy is essential for optimizing treatment outcomes. Further research in this area can significantly enhance the effectiveness of personalized cancer treatments.

To further investigate the differences in neoadjuvant immunotherapy among various cancer types to guide clinical practice better. We analyzed the five cancer types with the highest frequency of keywords. The development of neoadjuvant immunotherapy for NSCLC, breast cancer, bladder cancer, melanoma, and TNBC was compared. The different levels of development of neoadjuvant immunotherapy research in these cancer types are discussed, as well as the reasons for these differences.

Neoadjuvant immunotherapy in NSCLC has shown promising results. For instance, PD-1 blockade has successfully improved patient outcomes, with studies such as the SAKK 16/14 trial investigating the addition of durvalumab to neoadjuvant chemotherapy in stage IIIA (N2) NSCLC patients^33^. Combined therapies, such as nivolumab and ipilimumab, have also been explored in resectable NSCLC, with positive outcomes^34^. Furthermore, compartmental analysis of T-cell clonal dynamics has helped identify pathologic responses to neoadjuvant PD-1 blockade, providing insights into treatment efficacy and resistance^35^. Despite these advancements, further research is needed to optimize treatment strategies and improve patient outcomes.

Neoadjuvant immunotherapy for breast cancer has emerged as a promising approach, particularly in aggressive subtypes like TNBC. Studies have shown that neoadjuvant immunotherapy can improve outcomes compared to adjuvant therapy, which helps eradicate metastatic disease^23^. Combining ICIs, such as pembrolizumab and durvalumab, with standard anthracycline-taxane-based chemotherapy has shown clinical promise^36^. Research also reveals correlations between immune biomarkers, such as PD-L1 expression, TILs, and response to neoadjuvant therapy^37^. These findings highlight the therapeutic potential of neoadjuvant immunotherapy and emphasize the need for further research to optimize treatment strategies.

Developing neoadjuvant immunotherapy in bladder cancer has centered around using ICIs, such as pembrolizumab and atezolizumab. The PURE-01 study demonstrated the preliminary activity of neoadjuvant pembrolizumab in patients with muscle-invasive bladder carcinoma^38^. At the same time, the NABUCCO trial investigated the preoperative combination of ipilimumab and nivolumab in locoregionally advanced urothelial cancer^39^. Although the FDA has approved atezolizumab for treating progressive advanced urothelial carcinoma, more research is needed to explore the full potential of neoadjuvant immunotherapy in this cancer type.

Neoadjuvant immunotherapy has shown significant promise in melanoma, particularly in combination therapies such as ipilimumab and nivolumab. The OpACIN-neo and OpACIN trials analyzed survival and biomarker data from neoadjuvant immunotherapy in stage III melanoma patients^1^. Additionally, immune circulation and tumor microenvironment monitoring has been investigated in patients with regionally advanced melanoma receiving neoadjuvant ipilimumab^40^. While these studies provide valuable insights into treatment efficacy and patient stratification, further research is necessary to refine neoadjuvant immunotherapy approaches in melanoma.

TNBC, a particularly aggressive breast cancer subtype, has seen significant research into neoadjuvant immunotherapy. Studies such as the GeparNuevo trial have investigated the use of durvalumab in addition to anthracycline-taxane-based neoadjuvant therapy, with promising results^36^. Another area of interest has been the relationship between RAS/MAPK activation and TILs and the therapeutic cooperation between MEK inhibitors and PD-1/PD-L1 ICIs^22^. The role of PD-L1 expression and TILs in TNBC prognosis has also been investigated, highlighting the importance of understanding the tumor microenvironment in developing effective immunotherapies^41^.

Innovative approaches, such as transformable nanoparticle-enabled immunotherapy and anti-folate receptor alpha-directed antibody therapies, have been explored to enhance TNBC treatment^42^. Furthermore, neoadjuvant interferons are critical for effective PD-1-based immunotherapy in TNBC^43^. Lastly, the therapeutic cooperation between auranofin, a thioredoxin reductase inhibitor, and anti-PD-L1 antibodies has been examined for TNBC treatment^44^.

The development of neoadjuvant immunotherapy varies across cancer types, with melanoma and NSCLC demonstrating more advanced progress than bladder, breast, and TNBC. These disparities may include differences in tumor biology, the immune microenvironment, and the prevalence of specific cancer subtypes that respond well to immunotherapies.

Melanoma, in particular, has shown a high response rate to immunotherapies due to its high mutational burden and immunogenicity^45^. NSCLC also exhibits significant progress in neoadjuvant immunotherapy development, partly because of its high incidence and the presence of actionable molecular targets, such as PD-L1^46^.

In contrast, bladder cancer, breast cancer, and TNBC have experienced a slower pace of development in neoadjuvant immunotherapy. The complex tumor biology and heterogeneity of breast cancer, including varying responses to treatment across subtypes, may partly explain this disparity. TNBC, a highly aggressive subtype of breast cancer, has shown promise in neoadjuvant immunotherapy research, but challenges remain in understanding and overcoming immune evasion mechanisms. Lastly, bladder cancer has a lower incidence than NSCLC and breast cancer, which may contribute to the slower progress in neoadjuvant immunotherapy research.

Neoadjuvant immunotherapy has demonstrated the potential to improve patient outcomes across various cancer types, including NSCLC, breast cancer, bladder cancer, melanoma, and TNBC. While melanoma and NSCLC have made significant progress, bladder cancer, breast cancer, and TNBC still require further investigation to optimize treatment strategies. Understanding the underlying reasons for disparities in neoadjuvant immunotherapy development can help guide future research, with the ultimate goal of providing personalized and effective treatment options for patients.

CiteSpace’s ‘burst detection’ method identifies keywords or cited references with significant changes over time^9^. Researchers can use keywords and cited references with burst features to explore hotspots of research. In this study, ‘urothelial carcinoma’, ‘ipilimumab’, and ‘radical cystectomy’ were keywords that continued to burst as of 2022. This suggests that neoadjuvant immunotherapy for urological system cancer may be a potential research hotspot in the future^39,47–49^. In addition, seven cited references continued to burst in 2022. Of these, three studies focused on the application of ICIs in neoadjuvant immunotherapy^14,15,23^, and two discussed the application of ICIs in adjuvant therapy^50,51^. One study by Necchi *et al*. is worth noting. This study showed that pembrolizumab could be a reliable neoadjuvant therapy for MIBC, limited to patients with PD-L1 positivity or high tumor mutation burden. This is consistent with the results of the burst detection of keywords^18^. In addition, Ayers *et al*. reported an IFN-γ-related mRNA profile that can predict the efficacy of ICIs^52^. Reck *et al*.^53^ pointed out that in advanced NSCLC patients, pembrolizumab significantly prolonged progression-free survival and overall survival with fewer adverse events compared to platinum-based chemotherapy when PD-L1 was expressed in at least 50% of tumor cells. In light of the burst detection results mentioned above, clinical trials of neoadjuvant immunotherapy and predictive biomarkers for therapeutic efficacy in different types of cancer may be research directions worth paying attention to soon.

There are some limitations to this study. First, it only includes articles written in English and recorded in the WoSCC database. Since WoSCC covers the vast majority of high-quality studies, this does not affect the overall trend of the results. Second, recently published high-quality studies may not have received the attention they deserved due to citation delays and need to be updated in subsequent studies. Nevertheless, this study will significantly assist relevant researchers in understanding the development, hotspots, trends, and frontiers of neoadjuvant immunotherapy and identifying areas that still require further research.

## Conclusion

In recent years, there has been increasing attention paid to research related to neoadjuvant immunotherapy. The substantial increase in publications each year indicates the growing importance of this research field. This study identifies the top researchers and institutions worldwide involved in neoadjuvant immunotherapy research. Frontiers in Oncology is the most active journal, and Francesco Montorsi is the most influential author. Immunotherapy as a neoadjuvant treatment for urological cancer and the development of efficacy predictive biomarkers are hot topics. The long-term prognosis of patients receiving neoadjuvant immunotherapy may be a critical area for future research. Therefore, a comprehensive overview of the evolution and frontiers of the field is available to researchers and policymakers who are new to the area.

## Ethical approval

No ethical approval and patient consent were required for all analyses were based on literature research.

## Sources of funding

This research was partly supported by the National High Level Hospital Clinical Research Funding (2022-PUMCH-C-049).

## Author contribution

X.L., Y.X., and X.S.: conceived and designed the study; S.J.: wrote the manuscript and participated in the design of the study; S.J., H.Z., and Y.L.: were responsible for analysis and network construction; L.Z.: and performed the data analysis and data interpretation. All authors contributed to the article and reviewed the submitted version.

## Conflicts of interest disclosure

The authors declare that the research was conducted without any commercial or financial relationships that could be construed as a potential conflict of interest.

## Research registration Unique Identifying number (UIN)

Not required.

## Guarantor

Yiyao Xu, Department of Liver Surgery, Peking Union Medical College (PUMC) Hospital, PUMC & Chinese Academy of Medical Sciences, Dongcheng, Beijing 100730, China. E-mail: xuyiyao@pumch.cn.

Xin Lu, Department of Liver Surgery, Peking Union Medical College (PUMC) Hospital, PUMC & Chinese Academy of Medical Sciences, Dongcheng, Beijing 100730, China. E-mail: luxin@pumch.cn.

## Data availability statement

The data in this study is not sensitive in nature and is accessible in the public domain. The data is therefore available and not of a confidential nature. All the data used in the study have been included in the article/Supplementary Material.

## Supplementary Material

SUPPLEMENTARY MATERIAL
