# Supplementary material for: Evolutionary patterns and research frontiers in neoadjuvant immunotherapy: a bibliometric analysis
Source: Int J Surg. 2023 May 20;109(9):2774–83. doi: 10.1097/JS9.0000000000000492 (PMC10498839; doi:10.1097/JS9.0000000000000492)
Supplement: SUPPLEMENTARY MATERIAL [file js9-109-2774-s001.docx]

| **Keyword** | **Frequency** | **Search Strategy** | **Number of articles** |
| --- | --- | --- | --- |
| **Non-small cell lung cancer** | 93 | (TS = (Non-small cell lung cancer)) AND (TS = (Neoadjuvant)) AND (TS = (Immunotherapy)) OR (TS = (Immunotherapies)) | 188 |
| **Breast cancer** | 66 | (TS = (Breast cancer)) AND (TS = (Neoadjuvant)) AND (TS = (Immunotherapy)) OR (TS = (Immunotherapies)) | 238 |
| **Bladder cancer** | 41 | (TS = (Bladder cancer)) AND (TS = (Neoadjuvant)) AND (TS = (Immunotherapy)) OR (TS = (Immunotherapies)) | 129 |
| **Melanoma** | 38 | (TS = (Melanoma)) AND (TS = (Neoadjuvant)) AND (TS = (Immunotherapy)) OR (TS = (Immunotherapies)) | 175 |
| **Triple-negative breast cancer** | 38 | (TS = (Triple-negative breast cancer)) AND (TS = (Neoadjuvant)) AND (TS = (Immunotherapy)) OR (TS = (Immunotherapies)) | 84 |

**Table S2.** Top 5 cancer types with keyword frequency and search strategy.
