# Supplementary material for: Evolutionary patterns and research frontiers in neoadjuvant immunotherapy: a bibliometric analysis
Source: Int J Surg. 2023 May 20;109(9):2774–83. doi: 10.1097/JS9.0000000000000492 (PMC10498839; doi:10.1097/JS9.0000000000000492)
Supplement: SUPPLEMENTARY MATERIAL [file js9-109-2774-s002.docx]

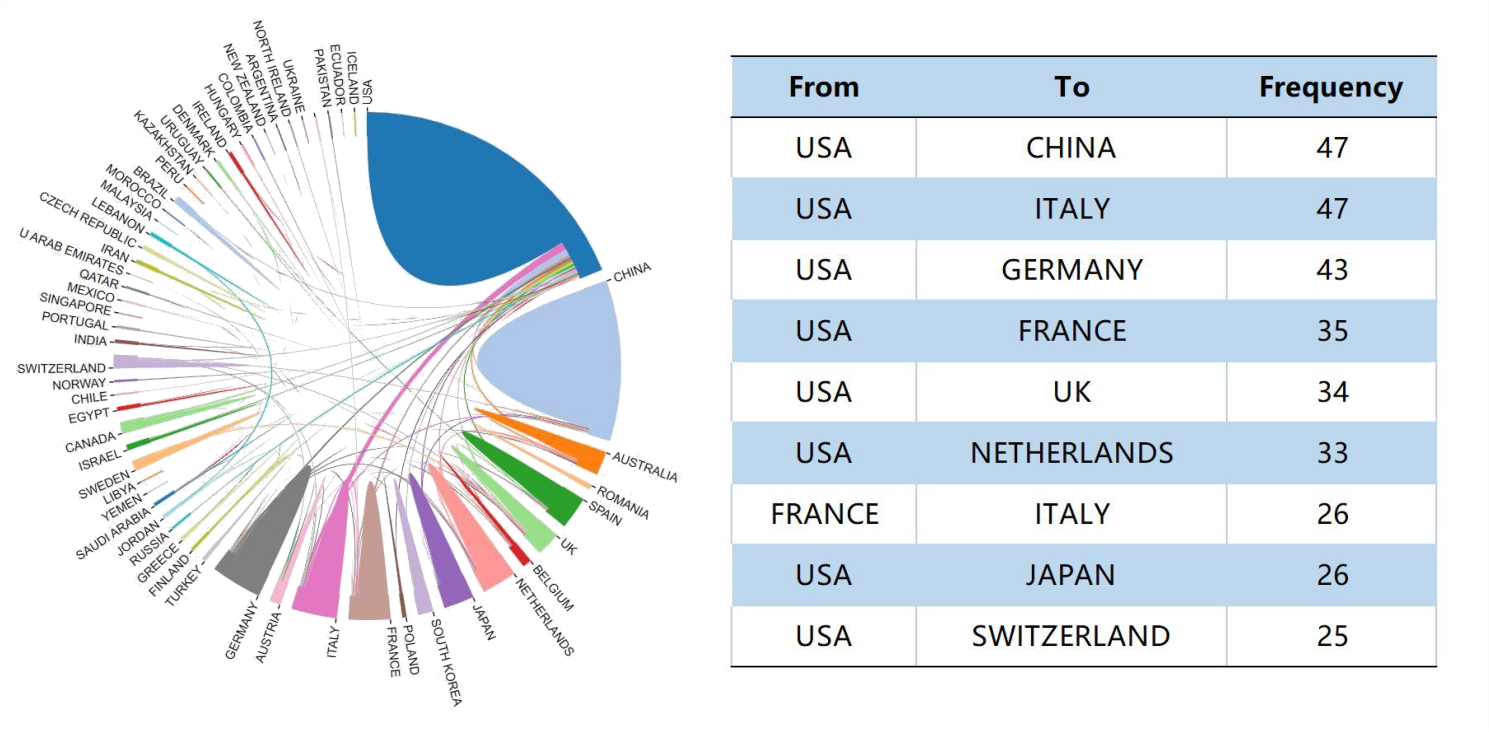


Figure S1. Global Collaborations in Neoadjuvant Immunotherapy and the Frequency of Collaboration Among Nations.


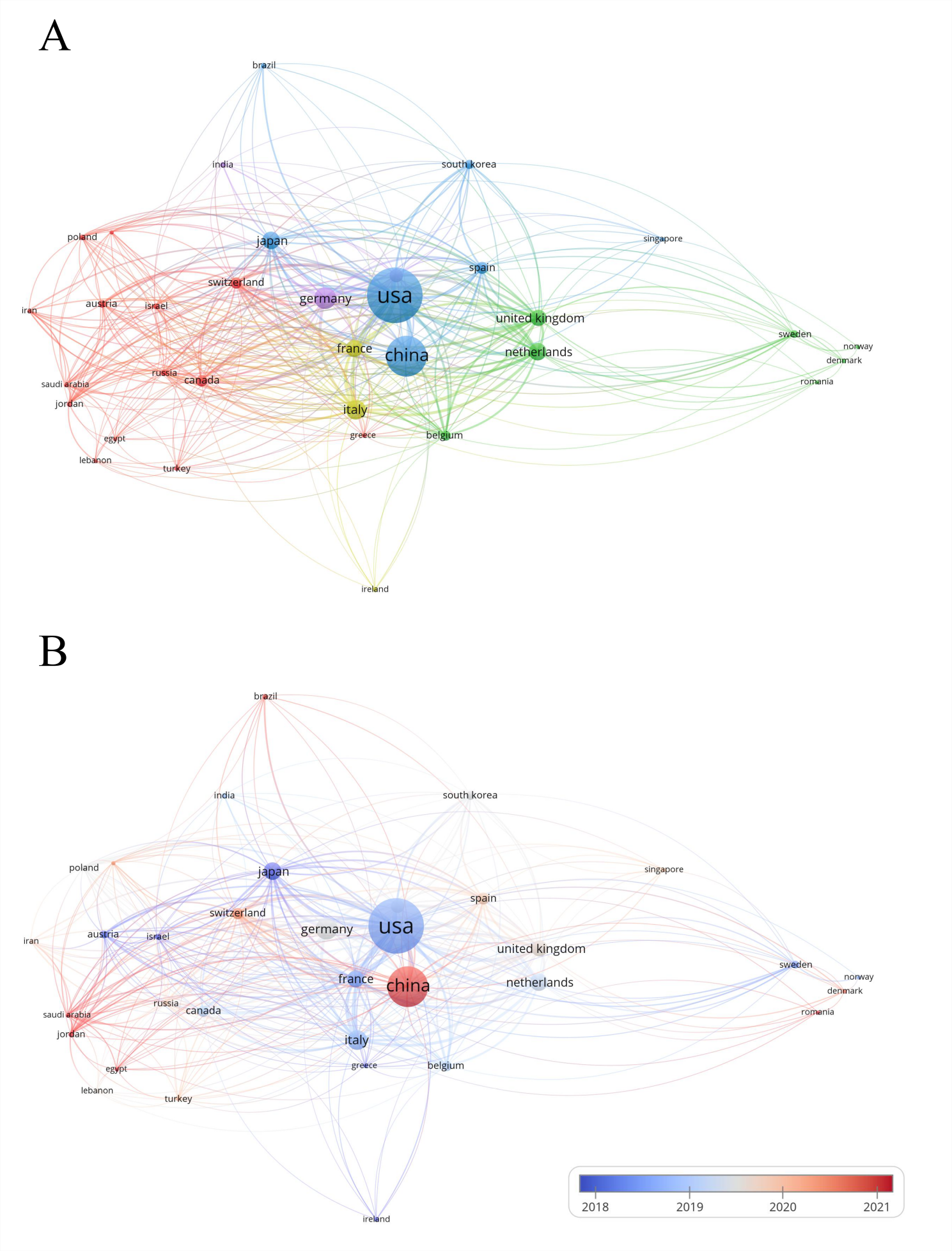


Figure S2. Graphical Representation of Country Co-authorship Analysis (A. Network Clustering; B. Time-Overlapping Network).


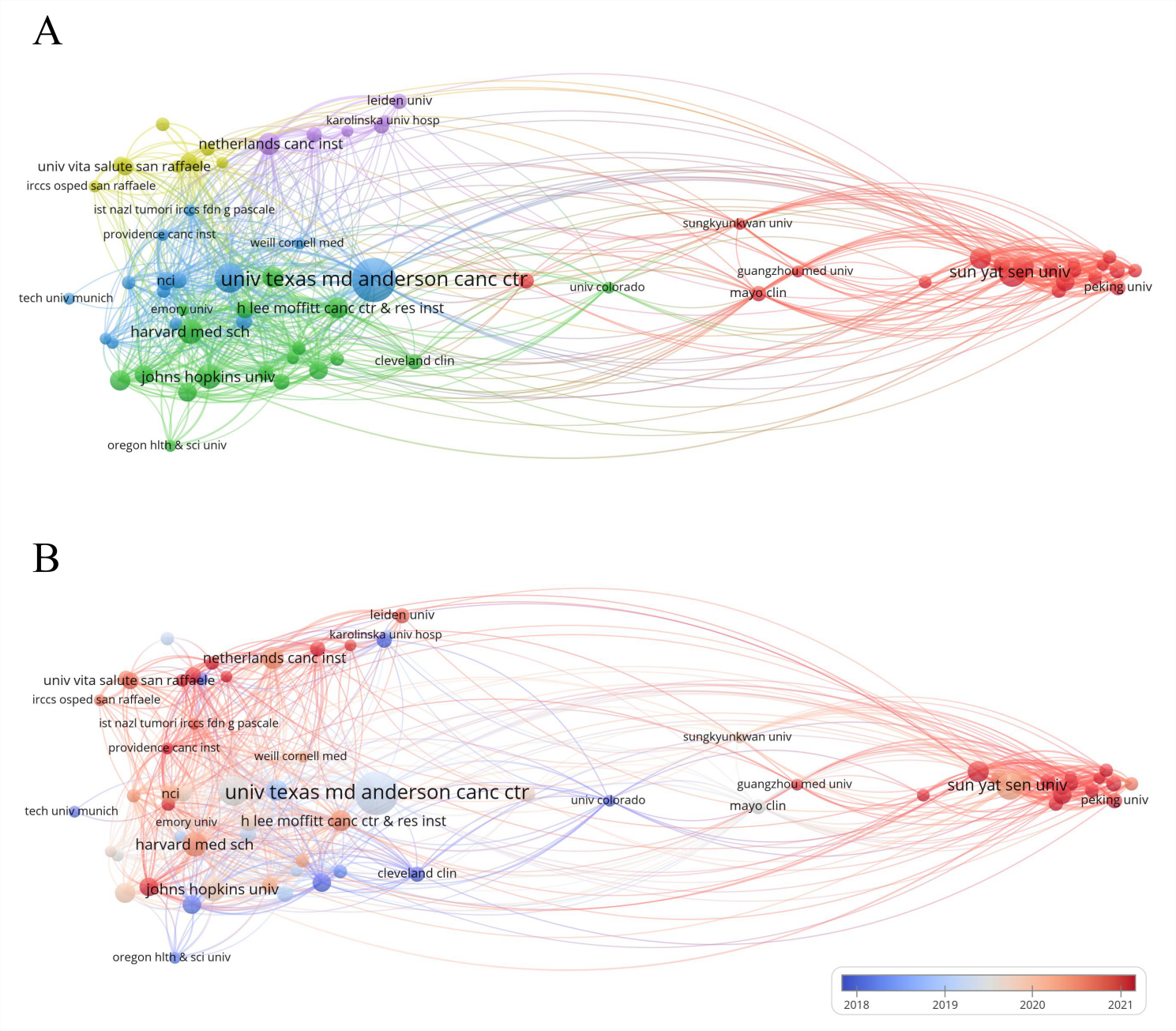


Figure S3. Graphical Representation of Institutional Co-authorship Analysis (A. Network Clustering; B. Time-Overlapping Network).


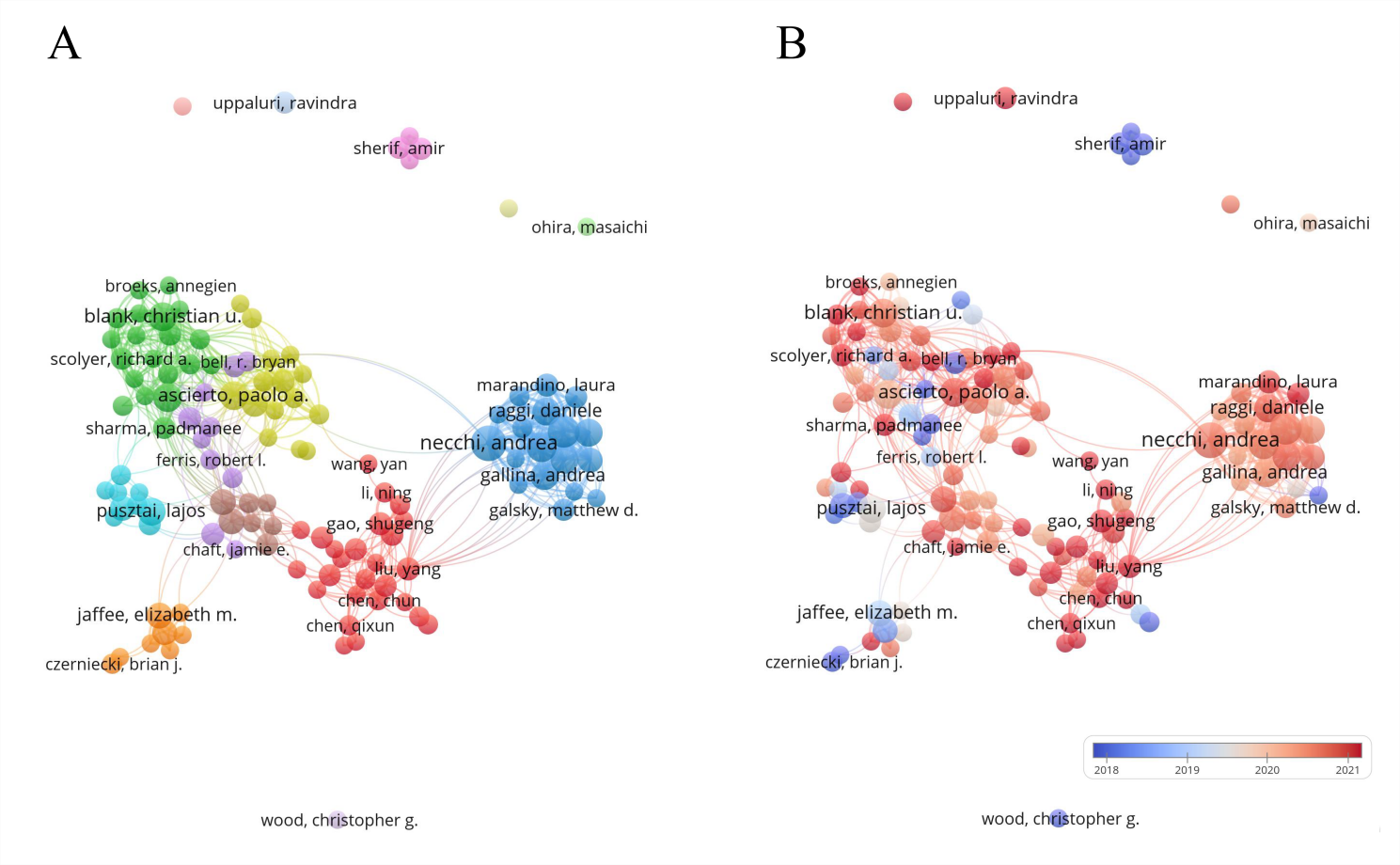


Figure S4. Graphical Representation of Researcher Co-authorship Analysis (A. Network Clustering; B. Time-Overlapping Network).


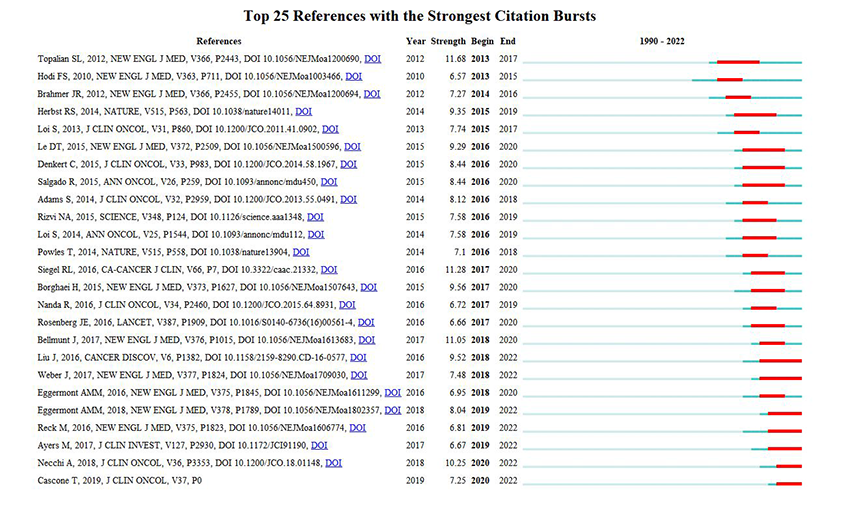


Figure S5. Top 25 Most Frequently Cited References in the Field of Neoadjuvant Immunotherapy.
