# Supplementary material for: Evolutionary patterns and research frontiers in neoadjuvant immunotherapy: a bibliometric analysis
Source: Int J Surg. 2023 May 20;109(9):2774–83. doi: 10.1097/JS9.0000000000000492 (PMC10498839; doi:10.1097/JS9.0000000000000492)
Supplement: SUPPLEMENTARY MATERIAL [file js9-109-2774-s004.docx]

**Table S4.** The top 10 cited articles in the neoadjuvant immunotherapy of non-small cell lung cancer.

| **Rank** | **Title** | **Year, Journal** | **Total citations** |
| --- | --- | --- | --- |
| 1 | Neoadjuvant PD-1 Blockade in Resectable Lung Cancer | 2018, The New England Journal of Medicine | 1031 |
| 2 | Predicting response to cancer immunotherapy using noninvasive radiomic biomarkers | 2019, Annals of Oncology | 231 |
| 3 | Initial results of pulmonary resection after neoadjuvant nivolumab in patients with resectable non-small cell lung cancer | 2019, The Journal of Thoracic and Cardiovascular Surgery | 139 |
| 4 | Toward personalized treatment approaches for non-small-cell lung cancer | 2021, Nature Medicine | 121 |
| 5 | Multispectral imaging for quantitative and compartment-specific immune infiltrates reveals distinct immune profiles that classify lung cancer patients | 2018, The Journal of Pathology | 102 |
| 6 | Tumor-infiltrating lymphocytes predict response to chemotherapy in patients with advance non-small cell lung cancer | 2012, Cancer Immunology and Immunotherapy | 98 |
| 7 | SAKK 16/14: Durvalumab in Addition to Neoadjuvant Chemotherapy in Patients With Stage IIIA(N2) Non-Small-Cell Lung Cancer-A Multicenter Single-Arm Phase II Trial | 2021, Journal of Clinical Oncology | 79 |
| 8 | Neoadjuvant nivolumab plus ipilimumab in resectable non-small cell lung cancer | 2020, The Journal for ImmunoTherapy of Cancer | 62 |
| 9 | Compartmental Analysis of T-cell Clonal Dynamics as a Function of Pathologic Response to Neoadjuvant PD-1 Blockade in Resectable Non-Small Cell Lung Cancer | 2020, Clinical Cancer Research | 61 |
| 10 | Alteration of PD-L1 expression and its prognostic impact after concurrent chemoradiation therapy in non-small cell lung cancer patients | 2017, Scientific Reports | 51 |
