# Supplementary material for: Evolutionary patterns and research frontiers in neoadjuvant immunotherapy: a bibliometric analysis
Source: Int J Surg. 2023 May 20;109(9):2774–83. doi: 10.1097/JS9.0000000000000492 (PMC10498839; doi:10.1097/JS9.0000000000000492)
Supplement: SUPPLEMENTARY MATERIAL [file js9-109-2774-s005.docx]

**Table S5.** The top 10 cited articles in the neoadjuvant immunotherapy of breast cancer.

| **Rank** | **Title** | **Year, Journal** | **Total citations** |
| --- | --- | --- | --- |
| 1 | Pembrolizumab for Early Triple-Negative Breast Cancer | 2020, The New England Journal of Medicine | 916 |
| 2 | Improved Efficacy of Neoadjuvant Compared to Adjuvant Immunotherapy to Eradicate Metastatic Disease | 2016, Cancer Discovery | 430 |
| 3 | RAS/MAPK Activation Is Associated with Reduced Tumor-Infiltrating Lymphocytes in Triple-Negative Breast Cancer: Therapeutic Cooperation Between MEK and PD-1/PD-L1 Immune Checkpoint Inhibitors | 2016, Clinical Cancer Research | 348 |
| 4 | Patterns of Immune Infiltration in Breast Cancer and Their Clinical Implications: A Gene-Expression-Based Retrospective Study | 2016, Plos Medicine | 334 |
| 5 | A randomised phase II study investigating durvalumab in addition to an anthracycline taxane-based neoadjuvant therapy in early triple-negative breast cancer: clinical results and biomarker analysis of GeparNuevo study | 2019, Annals of Oncology | 308 |
| 6 | PD-L1 Expression Correlates with Tumor-Infiltrating Lymphocytes and Response to Neoadjuvant Chemotherapy in Breast Cancer | 2015, Cancer Immunology Research | 264 |
| 7 | B7-H4 is highly expressed in ductal and lobular breast cancer | 2005, Clinical Cancer Research | 183 |
| 8 | Molecular Pathways: Involvement of Immune Pathways in the Therapeutic Response and Outcome in Breast Cancer | 2013, Clinical Cancer Research | 153 |
| 9 | Immune Checkpoint Inhibition Overcomes ADCP-Induced Immunosuppression by Macrophages | 2018, Cell | 136 |
| 10 | Tumor-infiltrating lymphocyte composition, organization and PD-1/PD-L1 expression are linked in breast cancer | 2017, Oncoimmunology | 134 |
