# Supplementary material for: Evolutionary patterns and research frontiers in neoadjuvant immunotherapy: a bibliometric analysis
Source: Int J Surg. 2023 May 20;109(9):2774–83. doi: 10.1097/JS9.0000000000000492 (PMC10498839; doi:10.1097/JS9.0000000000000492)
Supplement: SUPPLEMENTARY MATERIAL [file js9-109-2774-s006.docx]

**Table S6.** The top 10 cited articles in the neoadjuvant immunotherapy of bladder cancer.

| **Rank** | **Title** | **Year, Journal** | **Total citations** |
| --- | --- | --- | --- |
| 1 | Treatment of muscle-invasive and advanced bladder cancer in 2020 | 2020, CA: A Cancer Journal for Clinicians | 288 |
| 2 | Updated Results of PURE-01 with Preliminary Activity of Neoadjuvant Pembrolizumab in Patients with Muscle-invasive Bladder Carcinoma with Variant Histologies | 2020, European Urology | 164 |
| 3 | Preoperative ipilimumab plus nivolumab in locoregionally advanced urothelial cancer: the NABUCCO trial | 2020, Nature Medicine | 133 |
| 4 | Neoadjuvant PD-L1 plus CTLA-4 blockade in patients with cisplatin-ineligible operable high-risk urothelial carcinoma | 2020, Nature Medicine | 112 |
| 5 | Siglec15 shapes a non-inflamed tumor microenvironment and predicts the molecular subtype in bladder cancer | 2021, Theranostics | 98 |
| 6 | FDA Approval Summary: Atezolizumab for the Treatment of Patients with Progressive Advanced Urothelial Carcinoma after Platinum-Containing ChemotherapyGeparNuevo study | 2017, The Oncologist | 87 |
| 7 | Landmarks in the treatment of muscle-invasive bladder cancer | 2017, Nature Reviews Urology | 84 |
| 8 | NCCN Guidelines (R) Insights Bladder Cancer, Version 2.2016 Featured Updates to the NCCN Guidelines | 2016, Journal of the National Comprehensive Cancer Network | 74 |
| 9 | Therapeutic approaches to bladder cancer: identifying targets and mechanisms | 2003, Critical Reviews in Oncology/Hematology | 39 |
| 10 | Immunotherapy for Urothelial Carcinoma: Current Evidence and Future Directions | 2018, Current Urology Reports | 37 |
