# Supplementary material for: Evolutionary patterns and research frontiers in neoadjuvant immunotherapy: a bibliometric analysis
Source: Int J Surg. 2023 May 20;109(9):2774–83. doi: 10.1097/JS9.0000000000000492 (PMC10498839; doi:10.1097/JS9.0000000000000492)
Supplement: SUPPLEMENTARY MATERIAL [file js9-109-2774-s007.docx]

**Table S7.** The top 10 cited articles in the neoadjuvant immunotherapy of melanoma.

| **Rank** | **Title** | **Year, Journal** | **Total citations** |
| --- | --- | --- | --- |
| 1 | B cells and tertiary lymphoid structures promote immunotherapy response | 2020, Nature | 905 |
| 2 | Improved Efficacy of Neoadjuvant Compared to Adjuvant Immunotherapy to Eradicate Metastatic Disease | 2016, Cancer Discovery | 430 |
| 3 | Neoadjuvant versus adjuvant ipilimumab plus nivolumab in macroscopic stage III melanoma | 2018, Nature Medicine | 404 |
| 4 | Predicting response to cancer immunotherapy using noninvasive radiomic biomarkers | 2019, Annals of Oncology | 231 |
| 5 | Immune Monitoring of the Circulation and the Tumor Microenvironment in Patients with Regionally Advanced Melanoma Receiving Neoadjuvant Ipilimumab | 2014, Plos One | 215 |
| 6 | Preoperative ipilimumab plus nivolumab in locoregionally advanced urothelial cancer: the NABUCCO trial | 2020, Nature Medicine | 133 |
| 7 | Pathological response and survival with neoadjuvant therapy in melanoma: a pooled analysis from the International Neoadjuvant Melanoma Consortium (INMC) | 2021, Nature Medicine | 128 |
| 8 | Mechanisms of immune evasion in breast cancer | 2018, BMC Cancer | 121 |
| 9 | Survival and biomarker analyses from the OpACIN-neo and OpACIN neoadjuvant immunotherapy trials in stage III melanoma | 2021, Nature Medicine | 107 |
| 10 | Identification of genetic determinants of breast cancer immune phenotypes by integrative genome-scale analysis | 2017, Oncoimmunology | 107 |
