# Supplementary material for: Evolutionary patterns and research frontiers in neoadjuvant immunotherapy: a bibliometric analysis
Source: Int J Surg. 2023 May 20;109(9):2774–83. doi: 10.1097/JS9.0000000000000492 (PMC10498839; doi:10.1097/JS9.0000000000000492)
Supplement: SUPPLEMENTARY MATERIAL [file js9-109-2774-s008.docx]

**Table S8.** The top 10 cited articles in the neoadjuvant immunotherapy of triple-negative breast cancer.

| **Rank** | **Title** | **Year, Journal** | **Total citations** |
| --- | --- | --- | --- |
| 1 | Pembrolizumab for Early Triple-Negative Breast Cancer | 2020, The New England Journal of Medicine | 916 |
| 2 | RAS/MAPK Activation Is Associated with Reduced Tumor-Infiltrating Lymphocytes in Triple-Negative Breast Cancer: Therapeutic Cooperation Between MEK and PD-1/PD-L1 Immune Checkpoint Inhibitors | 2016, Clinical Cancer Research | 348 |
| 3 | A randomised phase II study investigating durvalumab in addition to an anthracycline taxane-based neoadjuvant therapy in early triple-negative breast cancer: clinical results and biomarker analysis of GeparNuevo study | 2019, Annals of Oncology | 308 |
| 4 | Molecular Pathways: Involvement of Immune Pathways in the Therapeutic Response and Outcome in Breast Cancer | 2013, Clinical Cancer Research | 153 |
| 5 | A single-cell map of intratumoral changes during anti-PD1 treatment of patients with breast cancer | 2021, Nature Medicine | 130 |
| 6 | The combination of PD-L1 expression and decreased tumor-infiltrating lymphocytes is associated with a poor prognosis in triple-negative breast cancer | 2017, Oncotarget | 87 |
| 7 | Transformable Nanoparticle-Enabled Synergistic Elicitation and Promotion of Immunogenic Cell Death for Triple-Negative Breast Cancer Immunotherapy | 2019, Advanced Functional Materials | 54 |
| 8 | Anti-Folate Receptor Alpha-Directed Antibody Therapies Restrict the Growth of Triple-negative Breast Cancer | 2018, Clinical Cancer Research | 49 |
| 9 | Neoadjuvant Interferons: Critical for Effective PD-1-Based Immunotherapy in TNBC | 2017, Cancer Immunology Research | 46 |
| 10 | Therapeutic cooperation between auranofin, a thioredoxin reductase inhibitor and anti-PD-L1 antibody for treatment of triple-negative breast cancer | 2020, International Journal of Cancer | 41 |
