# Supplementary material for: Evolutionary patterns and research frontiers in neoadjuvant immunotherapy: a bibliometric analysis
Source: Int J Surg. 2023 May 20;109(9):2774–83. doi: 10.1097/JS9.0000000000000492 (PMC10498839; doi:10.1097/JS9.0000000000000492)
Supplement: SUPPLEMENTARY MATERIAL [file js9-109-2774-s009.docx]

**Table S1.** The top 10 cited articles.

| **Rank** | **Title** | **Year, Journal** | **First author** | **Total citations** | **TC per Year** |
| --- | --- | --- | --- | --- | --- |
| 1 | Neoadjuvant PD-1 Blockade in Resectable Lung Cancer | 2018, The New England Journal of Medicine | Patrick Forde | 970 | 242.5 |
| 2 | Pembrolizumab for Early Triple-Negative Breast Cancer | 2020, The New England Journal of Medicine | Peter Schmid | 840 | 420 |
| 3 | B cells and tertiary lymphoid structures promote immunotherapy response | 2020, Nature | Beth Helmink | 837 | 418.5 |
| 4 | Low-Dose Irradiation Programs Macrophage Differentiation to an iNOS+/M1 Phenotype that Orchestrates Effective T Cell Immunotherapy | 2013, Cancer Cell | Felix Klug | 636 | 70.7 |
| 5 | Neoadjuvant anti-PD-1 immunotherapy promotes a survival benefit with intratumoral and systemic immune responses in recurrent glioblastoma | 2019, Nature Medicine | Timothy F. Cloughesy | 587 | 195.7 |
| 6 | Erdafitinib in Locally Advanced or Metastatic | 2019, The New England Journal of Medicine | Yohann Loriot | 563 | 187.7 |
| 7 | Improved Efficacy of Neoadjuvant Compared to Adjuvant Immunotherapy to Eradicate Metastatic Disease | 2016, Cancer Discovery | Jing Liu | 409 | 68.2 |
| 8 | Neoadjuvant versus adjuvant ipilimumab plus nivolumab in macroscopic stage III melanoma | 2018, Nature Medicine | Christian U Blank | 380 | 95 |
| 9 | Neoadjuvant immunotherapy leads to pathological responses in MMR-proficient and MMR-deficient early-stage colon cancers | 2020, Nature Medicine | Myriam Chalabi | 371 | 185.5 |
| 10 | RAS/MAPK Activation Is Associated with Reduced Tumor-Infiltrating Lymphocytes in Triple-Negative Breast Cancer: Therapeutic Cooperation Between MEK and PD-1/PD-L1 Immune Checkpoint Inhibitors | 2016, Clinical Cancer Research | Sherene Loi | 340 | 56.7 |
